# The plant toxin 4-methylsulfinylbutyl isothiocyanate decreases herbivore performance and modulates cellular and humoral immunity

**DOI:** 10.1371/journal.pone.0289205

**Published:** 2023-08-02

**Authors:** Enakshi Ghosh, Ghada S. Y. Tafesh-Edwards, Ioannis Eleftherianos, Stephanie L. Goldin, Paul J. Ode

**Affiliations:** 1 Department of Agricultural Biology, Colorado State University, Fort Collins, Colorado, Unites States of America; 2 Department of Biological Sciences, The George Washington University, Washington, D.C., Unites States of America; 3 Graduate Degree Program in Ecology, Colorado State University, Fort Collins, Colorado, Unites States of America; University of Arkansas, UNITED STATES

## Abstract

Insect herbivores frequently encounter plant defense molecules, but the physiological and ecological consequences for their immune systems are not fully understood. The majority of studies attempting to relate levels of plant defensive chemistry to herbivore immune responses have used natural population or species-level variation in plant defensive chemistry. Yet, this potentially confounds the effects of plant defense chemistry with other potential plant trait differences that may affect the expression of herbivore immunity. We used an artificial diet containing known quantities of a plant toxin (4-methylsulfinylbutyl isothiocyanate; 4MSOB-ITC or ITC, a breakdown product of the glucosinolate glucoraphanin upon herbivory) to explicitly explore the effects of a plant toxin on the cellular and humoral immune responses of the generalist herbivore *Trichoplusia ni* (Lepidoptera: Noctuidae) that frequently feeds on glucosinolate-containing plants. Caterpillars feeding on diets with high concentrations of ITC experienced reduced survivorship and growth rates. High concentrations of ITC suppressed the appearance of several types of hemocytes and melanization activity, which are critical defenses against parasitic Hymenoptera and microbial pathogens. In terms of *T*. *ni* humoral immunity, only the antimicrobial peptide (AMP) genes *lebocin* and *gallerimycin* were significantly upregulated in caterpillars fed on diets containing high levels of ITC relative to caterpillars that were provided with ITC-free diet. Surprisingly, challenging caterpillars with a non-pathogenic strain of *Escherichia coli* resulted in the upregulation of the AMP gene *cecropin*. Feeding on high concentrations of plant toxins hindered caterpillar development, decreased cellular immunity, but conferred mixed effects on humoral immunity. Our findings provide novel insights into the effects of herbivore diet composition on insect performance demonstrating the role of specific plant defense toxins that shape herbivore immunity and trophic interactions.

## Introduction

Plants have a diverse array of chemical defenses against insect herbivores. However, many herbivores have developed counter-defense mechanisms such as excretion, detoxification, and sequestration to overcome the negative effects of plant defense metabolites [1, 2]. This coevolutionary battle to eat or not to be eaten is one of the major drivers of speciation in both plants and insects [3]. Previous studies of plant-herbivore coevolution have shown that caterpillars possessing specialized mechanisms to deal with plant toxins can successfully develop on the host plant, while caterpillars without specialized counter-defense mechanisms mostly avoid or perform poorly on well defended host plants [4]. Typically, generalist herbivores lack specialized mechanisms to deal with plant metabolites [1]. However, some generalists adjust their feeding behavior to the available food plant by altering their midgut responses in a process known as ‘physiological remodeling’ [5]. However, physiological remodeling is a metabolically costly process [6].

Organisms that occupy a middle trophic level like caterpillars must balance the potentially conflicting bottom-up pressures of plant anti-herbivore defenses and top-down pressures from their own natural enemies [7]. Hence, along with the ability to cope with plant toxins, caterpillars must also defend against their natural enemies to successfully survive. Previous research has shown a direct correlation between plant toxins and caterpillar immune responses [8–10]. For example, plant defense metabolites like catalpol and other iridoid glycosides suppress the immunocompetence of sequestering species such as *Junonia coenia* (Lepidoptera: Nymphalidae) and *Ceratomia catalpa* (Lepidoptera: Sphingidae) [7, 11]. On the other hand, glucosinolates have been shown to enhance the immune system of specialist herbivores that detoxify plant toxins such as *Plutella xylostella* (Lepidoptera: Plutellidae) and *Pieris rapae* (Lepidoptera: Pieridae) [10, 12]. In addition, studies have demonstrated that pyrrolizidine alkaloids enhance the survival of *Grammia incorrupta* caterpillars against parasitoids [13]. Similarly, the consumption of an exotic plant, *Plantago lanceolata*, has been shown to enhance the cellular immunity of *Anartia jatrophae* against pathogens [14]. These contrasting findings prompted us to investigate how a generalist herbivore balances between detoxifying plant anti-herbivore defense toxins and maintaining a robust immunity to fight against natural enemies.

Caterpillars employ both cellular and humoral immune responses against invaders, such as parasitic wasps and microbial pathogens [15]. Previous studies of the relationship between plant toxins and caterpillar immunity have focused on the total number of hemocytes in circulation and/or the melanization capacity which are often inconclusive [16]. Insect cellular immune responses are regulated by four major types of hemocytes, each of which has specific functions: granulocytes are active against pathogens through nodulation, encapsulation, and phagocytosis; pro-hemocytes have a stem-cell-like nature; oenocytoids produce the phenoloxidase enzyme that produces melanin; and plasmatocytes are primarily involved in the encapsulation of parasitic wasp eggs [15, 17]. Humoral immunity involves mostly soluble factors such as antimicrobial peptides (AMPs), which are primarily expressed in the insect fat-body and are crucial for pathogen elimination [18–20]. Upon pathogen detection, the humoral immune response leads to the activation of distinct immune signaling pathways: the immune deficiency (IMD) pathway is mostly triggered by Gram-negative bacteria, and the Toll pathway is mainly initiated by Gram-positive bacteria and fungi. Although these immune pathways lead to the production of specific AMPs, certain AMPs such as cecropin, attacin, lebocin, gloverin, and gallerimycin are produced by both pathways. This redundancy in the immune response can confer a broad-spectrum defense against pathogens, ensuring that the insect is equipped with multiple mechanisms to combat a wide range of invading microorganisms [21, 22].

Both aspects of the innate immune system work to oppose foreign invaders. Hence, an understanding of caterpillar immune status requires the simultaneous study of both cellular and humoral immune functions. Furthermore, maintaining an elevated immune status is costly, thus caterpillars must devote nutrient resources towards immunity, which may compromise their ability to detoxify plant defensive chemistry [23]. For this reason, it is important to correlate how plant toxins affect the activity of caterpillar cellular and humoral immune reactions as well as caterpillar development in order to understand the effect of plant defense toxins on caterpillar performance. We hypothesize that the consumption of plant toxins by caterpillars affects their cellular and humoral immune functions, as well as their development. Specifically, we predict that the consumption of plant toxins will compromise caterpillar immune function and development as the caterpillars must divert nutrient resources towards maintaining elevated immunity. We also predict that the effects of plant toxins on caterpillar immune function will be complex, with some toxins enhancing immune response and others suppressing it. By studying both cellular and humoral immune mechanisms in caterpillars, and correlating these with caterpillar development, we aim to gain a better understanding of the trade-offs involved in the interaction between plant toxins and caterpillar immunity, and how these trade-offs ultimately affect caterpillar performance.

Here we studied *Trichoplusia ni* (Lepidoptera: Noctuidae), an herbivore with a broad host range, that commonly feeds on plants in the genus *Brassica*. *Brassica* produce glucosinolates as their anti-herbivore defense metabolites [24]. Following damage from chewing herbivores, glucosinolates undergo hydrolysis with the plant enzyme myrosinase, which leads to the production of biologically active derivatives including nitriles, thiocyanates, and most importantly isothiocyanates [25]. Isothiocyanates are considered highly toxic, especially to generalist herbivores like *T*. *ni* [26, 27]. Yet, previous studies have shown that, despite having a broad host range, *T*. *ni* prefers to oviposit and feed on glucosinolate containing host plants, even when given a choice [28] Hence, it is important to examine the physiological and immunological effects of glucosinolates and *T*. *ni*.

As different plant species vary in more than just defensive chemistry, studies comparing plant species effects on caterpillar immunity can only make correlative associations between plant chemical defenses and herbivore immune responses. To determine whether isothiocyanates directly affect *T*. *ni* larval performance and immune responses, we use artificial diets to which we added known quantities of 4-methylsulfinylbutyl isothiocyanate (4- MSOB -ITC or ITC). We examine how different concentrations of the isothiocyanate mixed in an artificial diet affects the weight, mortality, development, as well as cellular and humoral immune responses of *T*. *ni* caterpillars. Our study illustrates that certain plant toxins can strongly influence herbivore performance and modify innate immune capacity.

## Materials and methods

### Insect colonies

A laboratory colony of *T*. *ni* was maintained in an environmental chamber with conditions set at 25±5°C, a 16L:8D photoperiod, and 30% relative humidity for several generations on artificial diet before use in experiments following an established protocol [29] (S1). Briefly, adult *T*. *ni* were provided with 10% honey water solution as a food source and allowed to mate inside a plastic container (3.78 L) that was covered with a sheet of paper towel on which *T*. *ni* adults laid eggs. The egg sheet was collected the next day, it was placed in petri dishes, and incubated at 25°C for three days until the *T*. *ni* eggs hatched. After hatching, first instar *T*. *ni* larvae were used in all experiments.

### Isothiocyanate effects on caterpillar development

*Trichoplusia ni* caterpillars primarily feed on cabbage that contains glucosinolates, the most abundant of which (approximately 95%) are aliphatic compounds like sinigrin and glucoraphanin. Hydrolysis of this compound produces allyl-isothiocyanate. The concentration of allyl-isothiocyanate (allyl-ITC) in *Brassica* plants can reach up to 3 μmol/g [26]. Based on this information, our four experimental treatments were: 0 (control), 1, 2, and 3 μmol of 4-MSOB -ITC/g of diet (4-MSOB; Sigma-Aldrich; CAS Number: 142825-10-3). The artificial diet [29] used in these treatments differed only in the concentrations of 4- MSOB -ITC. Caterpillars were kept individually in 37 ml plastic cups (SOLO^®^ soufflé cups, Dart Container Corp.) on approximately 15 ml of artificial diet containing one of the four isothiocyanate concentrations (*n* = 30/treatment). After eleven days of feeding on their respective diet, caterpillars were weighed and returned to their cups. Caterpillars were maintained on the same diet until pupation when pupae were weighed (*n* = 22/treatment). Survival rates were measured by monitoring a separate group of caterpillars, which were reared under the same experimental conditions. The number of caterpillars that successfully pupated was recorded out of the initial number of first instars placed on each of the four treatment diets allowing us to calculate survival rates. (*n* = 210 caterpillars/treatment). The total development duration from egg to pupal stage was recorded (*n* = 21/treatment).

### Isothiocyanate effects on caterpillar immune status

*Cellular immunity* To quantify the effect of 4- MSOB -ITC on the cellular immune status of *T*. *ni*, 11-day-old larvae (between first to late second instar) were collected from each of the isothiocyanate treatments (*n* = 10/treatment). Using a sterile needle, a lateral incision was made across the abdomen of each larva and the hemolymph was collected and added to a pre-chilled Eppendorf tube. To quantify the total number of hemocytes, the collected hemolymph was diluted with a fixed amount of phosphate buffer saline (PBS, 1:2 hemolymph:saline). Approximately 8 μL of diluted hemolymph was added onto a Neubauer hemocytometer and left to settle for 15 minutes, after which individual hemocytes were counted under the microscope (Leica DM750, Germany) at 200x magnification. Individual hemocytes were identified morphologically by performing an FITC tagged phalloidin-DAPI staining using established protocols [30]. Briefly, approximately 10 μL of diluted hemolymph were added onto a Teflon-coated, pre-chilled slide and incubated for 30 minutes at 4°C. Hemocytes were fixed in 4% formaldehyde, followed by three washes in PBS, and stained using alexafluor 488 (1:2000, Thermo Fisher Scientific) for 6 hours. Images were recorded using a Zeiss LSM-800 confocal microscope from slides mounted with a DAPI-glycerol solution. Morphological identification of differential hemocytes count (DHC) was carried out as described by Gupta [31] (*n* = 10/treatment).

To measure the phenoloxidase activity, hemolymph from individual larvae was mixed with ice-cold PBS in a 1:2 ratio in an Eppendorf tube and centrifuged at 4000g for 15 min at 4°C. The supernatant was added to a micro plate well containing 20 μL of PBS and 50 μL of 2 mM L-Dopa as a substrate for phenoloxidase. The reaction was allowed to proceed for 30 minutes. The degree of melanization was recorded spectrophotometrically at 490 nm (Versamax multimode reader) (*n* = 10/treatment).

*Humoral immunity* To investigate the impact of ITC on *T*. *ni* humoral immunity, relative expression profiles of five AMP-encoding genes were quantified. We compared the AMP transcriptional expression profiles of unchallenged caterpillars to those challenged with the non-pathogenic K-12 strain of *Escherichia coli*. These experiments involved collection of hemolymph and fat body tissues from 11-day old caterpillars that had previously been fed on their respective diet. Also, caterpillars were injected with the *E*. *coli* strain K-12 (OD- 3 at 600 nm; 2.4 x 10^9^ cells) and then allowed to feed on their respective diet for 24 hours (*n* = 3/treatment). RNA was isolated by homogenization of samples in TRIzol reagent (Ambion, Life Technologies). RNA concentration and purity were measured using Nanodrop (A260/280 ~ 2). Reverse transcription was performed on 1 μg of total RNA using a High-Capacity cDNA Reverse Transcription Kit (Applied Biosystems). Quantitative PCR (qPCR) reactions were conducted and monitored with a CFX96 Real-Time System, C1000 Thermal Cycler (Bio-Rad) with the following cycling conditions: 95°C for 2 minutes, 40 repetitions of 95°C for 15 seconds followed by 61°C for 30 seconds, and then one round of 95°C for 15 seconds, 65°C for 5 seconds, and finally 95°C for 5 seconds. Each reaction well contained 10 μl of GreenLink No-ROX qPCR Mix (BioLink), 40 ng of cDNA template, forward and reverse primers (provided in S2) at a final concentration of 200 nM and ultrapure water to 20 μl total volume. A dissociation curve analysis was performed for all primer pairs, and all experimental samples yielded a single sharp peak at the amplicon’s melting temperature. All assays were run three times (three biological replicates, each representing the mRNA of a single caterpillar) and each experiments included technical replicates. Relative fold changes for each gene were set to 1 for the control treatment, which involved caterpillars kept on diet in the absence of ITC. For analysis, the comparative quantitation method (ΔΔCt) was used to contrast the different treatments, and transformed to absolute values with 2-ΔΔCt for obtaining relative fold changes.

### Statistical analysis

One-way analyses of variance (ANOVAs) were used to compare the different treatments, followed by Tukey’s post-hoc significance test at a level of 5%. Caterpillar survivorship was compared between the treatments with generalized linear model having binomial distribution with logit link function. All statistical analyses were performed using SPSS 28.0. Figures were made using Origin 2021b and BioRender software.

## Results

### Isothiocyanate effects on caterpillar development

**Fig 1:**
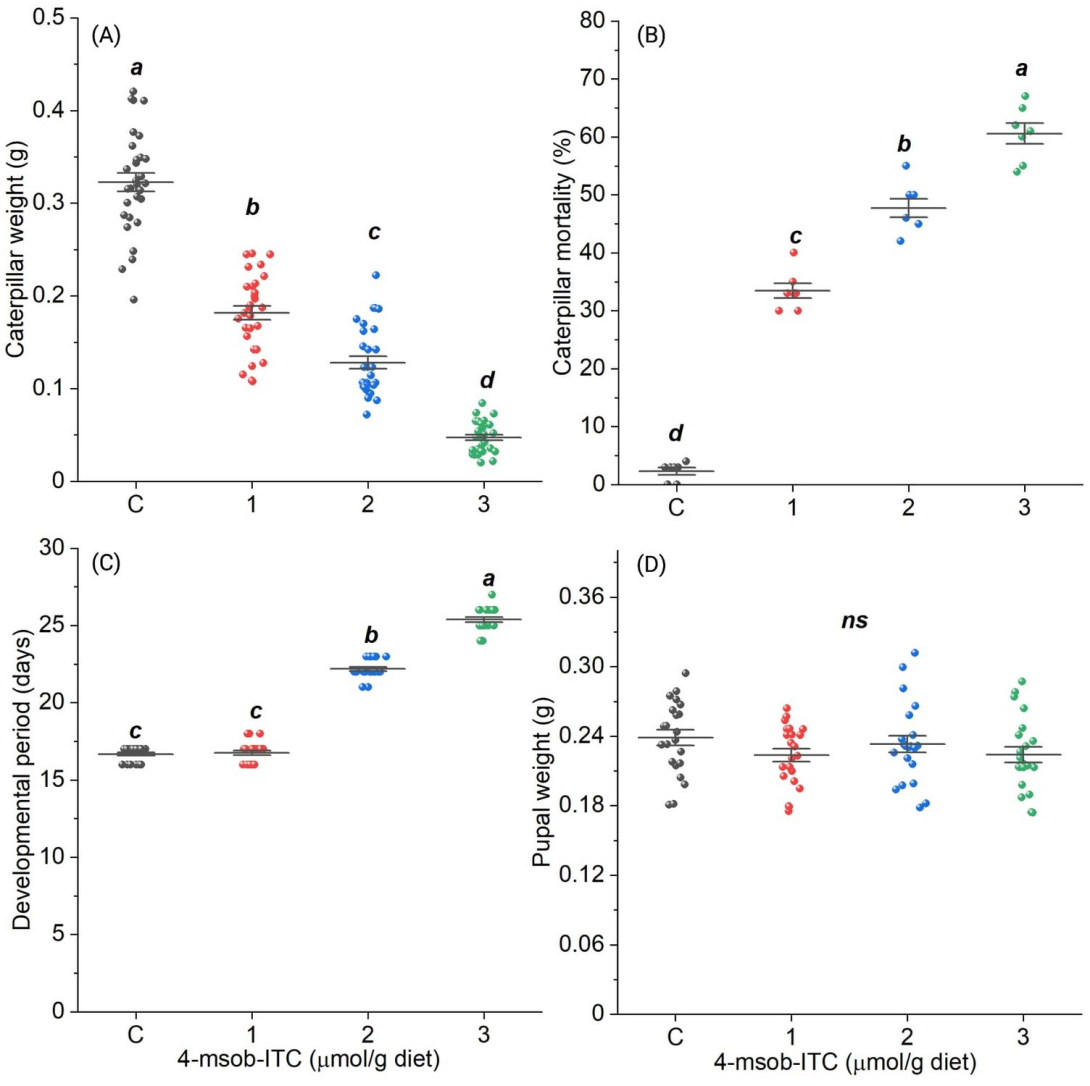
The effect of isothiocyanate (ITC) on the performance of *Trichoplusia ni*: (A) Caterpillar weight (g), (B) caterpillar mortality (%), (C) developmental duration (days), and (D) pupal weight (g). Values are means ± SE, significant differences are based on one-way ANOVA followed by Tukey’s post-hoc test at alpha level 0.05, caterpillar mortality was compared using generalized linear model.

Caterpillar performance was highest on an ITC-free diet (control), with minimal mortality and greater larval weight compared to caterpillars that fed on diets containing higher levels of ITC (Fig 1A–1C). Over an 11 day period, caterpillars fed diets containing 1, 2, or 3 μmol/g-ITC attained only 56%, 40%, or 15% of the weight of those fed on the ITC-free control diet (F_3,116_ = 251, P<0.001; Fig 1A). Caterpillar survival was reduced when insects were fed diets containing higher concentrations of ITC. While feeding on control diet, we observed only 2% caterpillar mortality, whereas mortality increased to 61% when caterpillars were fed on diets containing 3 μmol/g of ITC (*P*<0.001; Fig 1B). Also, caterpillars took 5.5 and 8 additional days to develop to pupation when they were fed on diets with 2 and 3 μmol/g-ITC, respectively (*P* = 0.008; Fig 1C). There was no significant difference in the duration of caterpillar development between the control treatment and the treatment containing diet supplemented with 1 μmol/g-ITC (*P* = 0.002; Fig 1C). Pupal weight was unaffected by the concentration of ITC in the diet (*F*_3,84_ = 1.24, *P* = 0.299; Fig 1D).

### Isothiocyanate effects on caterpillar cellular immunity

**Fig 2:**
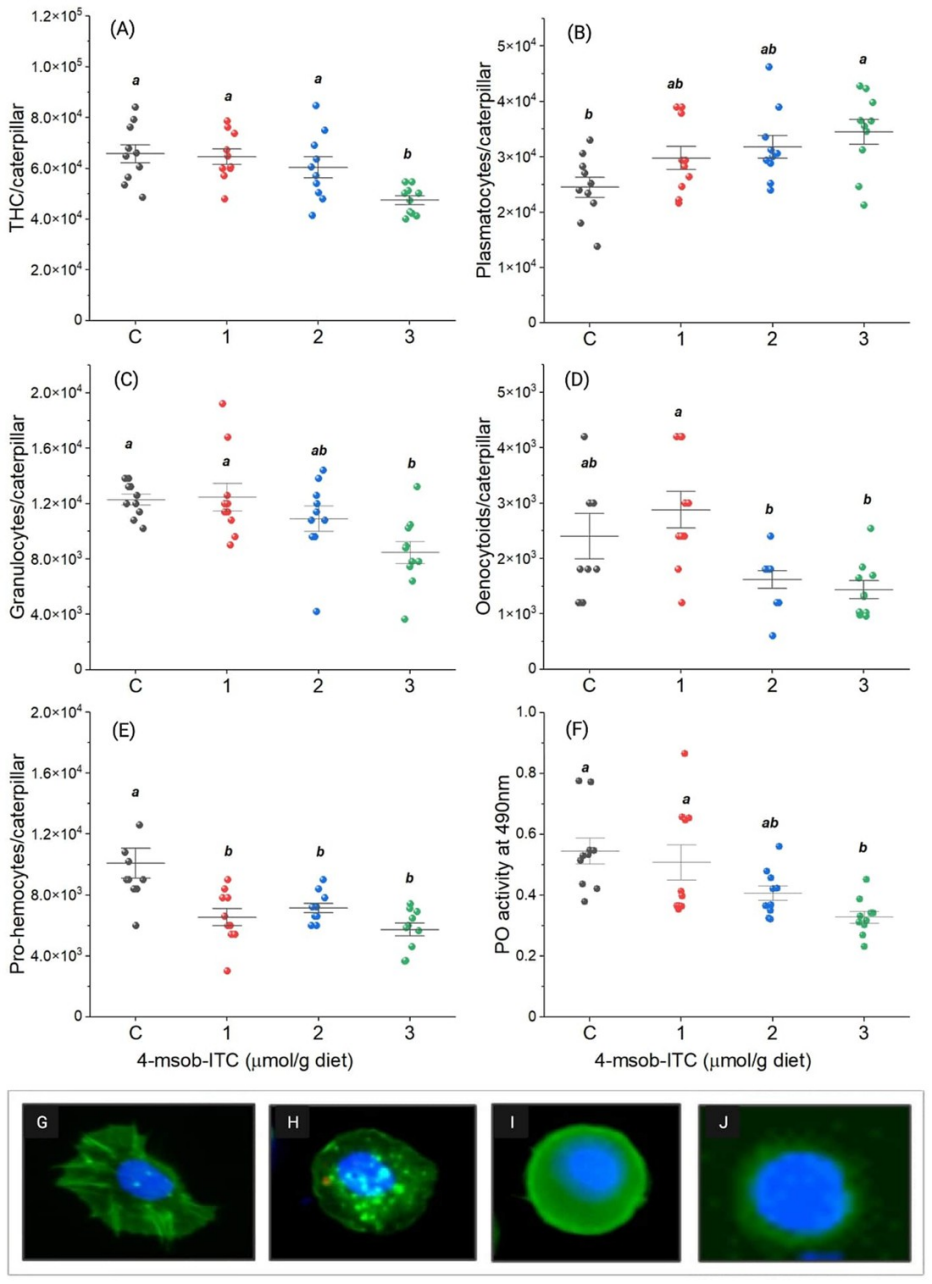
The effect of isothiocyanate (ITC) on the cellular immune response of *Trichoplusia ni*: (A) Total hemocyte count/caterpillar, (B) plasmatocyte numbers/caterpillar, (C) granulocyte numbers/caterpillar, (D) oenocytoid numbers/caterpillar, (E) pro-hemocyte numbers/caterpillar and (F) phenoloxidase (PO) activity at 490 nm, morphology of hemocytes recovered from *T*. *ni*: (G) plasmatocytes, (H) granulocytes, (I) oenocytoids, and (J) pro-hemocytes. Values are means ± SE, significant differences are based on one-way ANOVA followed by Tukey’s post-hoc test at alpha level 0.05.

The total hemocyte count in circulation decreased by 28% when caterpillars consumed diets containing 3 μmol/g of ITC compared to the control diet (*F*_3,36_ = 6.62, *P* = 0.001; Fig 2A). In general, numbers of different hemocyte types were also reduced when caterpillars were fed on diets containing higher levels of ITC. Granulocyte numbers were reduced by 31% when caterpillars were fed on diets containing 3 μmol/g ITC compared to control diets (*F*_3,36_ = 5.22, *P* = 0.004; Fig 2C and 2H). Similarly, oenocytoid numbers were reduced by 33% and 40% when the caterpillars consumed diet containing 2 and 3 μmol/g of ITC, respectively, (*F*_3,36_ = 5.56, *P* = 0.003; Fig 2D and 2I). Pro-hemocyte numbers were reduced by approximately 45% when caterpillars were fed on diets containing 1, 2, or 3 μmol/g of ITC compared to those fed on control diets (*F*_3,36_ = 9.2, *P* = 0.0001; Fig 2E and 2J). Furthermore, phenoloxidase activity, which represents the degree of the melanization response, was reduced by 40% in caterpillars fed on diet containing 3 μmol/g of ITC compared to individuals fed on control diet (*F*_3,36_ = 6.45, *P* = 0.001; Fig 2F). Interestingly, ITC concentration had a positive impact on plasmatocyte numbers as they increased by 18–30% in a dose dependent manner compared to the caterpillars kept on control diet (*F*_3,36_ = 3.2, *P* = 0.01; Fig 2B and 2G).

### Isothiocyanate effects on caterpillar humoral immunity

**Fig 3:**
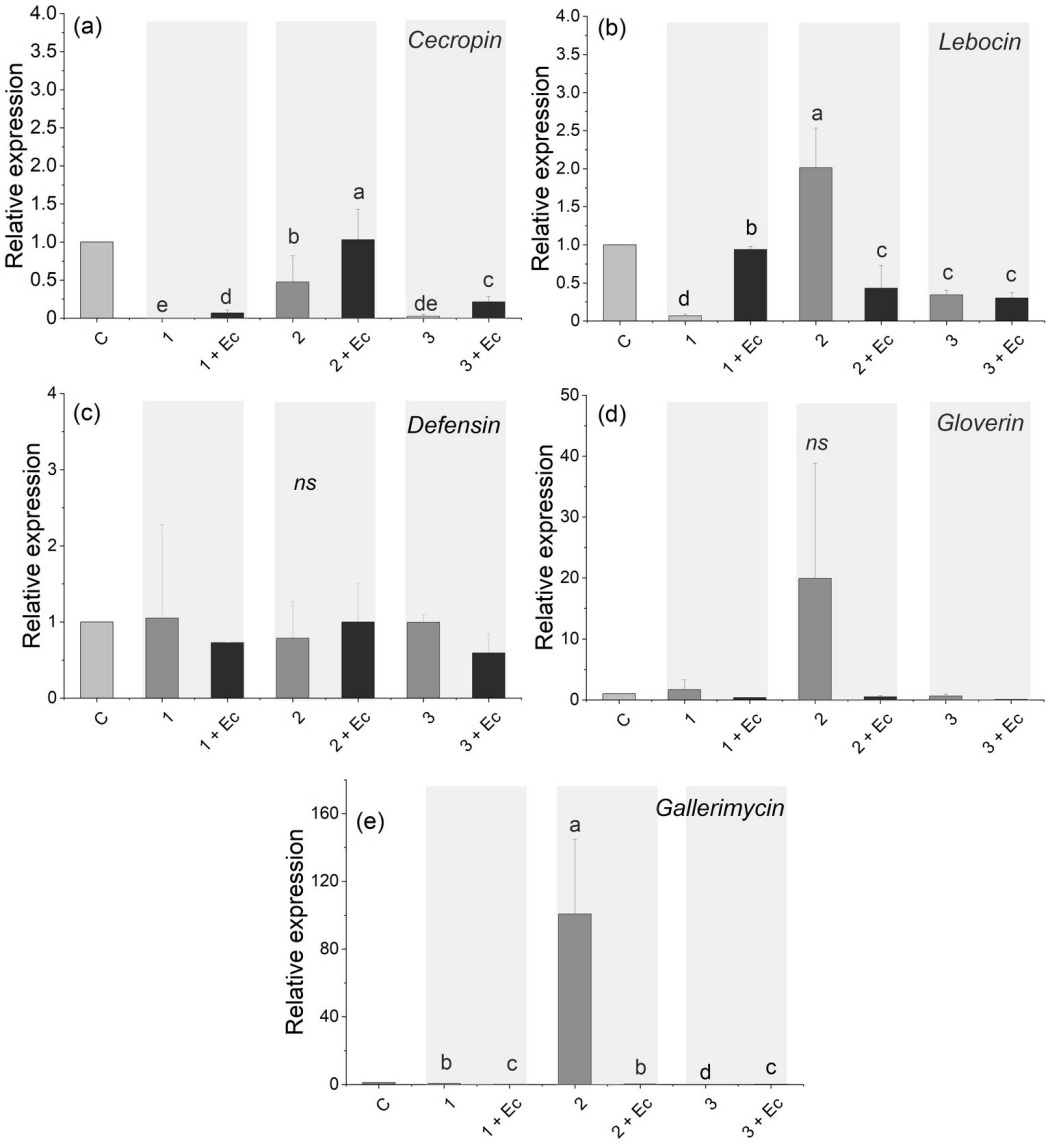
The effect of isothiocyanate (ITC) on the humoral immune response of *Trichoplusia ni*: Relative expression profile of (A) *cecropin*, (B) *lebocin*, (C) *defensin*, (D) *gloverin*, and (E) *gallerimycin* before and after *Escherichia coli* treatment. Values are means ± SE, significant differences are based on one-way ANOVA followed by Tukey’s post-hoc test at alpha level 0.05.

The relative fold change of five AMP-encoding genes at their basal level (uninduced) was compared and the expression two AMP genes, *lebocin* (*F*_5,12_ = 9, *P* = 0.001) and *gallerimycin* (*F*_*5*,12_ = 5.19, *P* = 0.009), was found to be significantly upregulated in caterpillars which had been fed on diet containing 2 μmol/g of ITC in comparison to control diet without ITC (Fig 3B and 3E). When caterpillars were challenged with *E*. *coli*, the expression of *cecropin* was upregulated compared to their uninfected controls (*F*_5,12_ = 3.94, *P* = 0.02; Fig 3A). *Lebocin* was upregulated only when caterpillars were fed on diet with 1 μmol/g-ITC, followed by *E*. *coli* challenge (*F*_5,12_ = 39, *P* = 0.0009; Fig 3B). No significant changes were found in *defensin* expression (*F*_5,12_ = 0.16, *P* = 0.9; Fig 3C). Interestingly, the increase in *gallerimycin* expression in uninfected caterpillars treated with 2 μmol/g of ITC was significantly decreased after infection with *E*. *coli* (*F*_*5*,12_ = 5.19, *P* = 0.009; Fig 3E).

## Discussion

Several studies show the correlation between total or individual glucosinolate levels with herbivore physiology and performance by using plant natural populations. These findings do not exclude the impact of other unquantified factors such as physical defenses, morphology, leaf toughness, and plant nutritional quality like protein, free amino acids, carbohydrates, antioxidants and various other macro- or micro-nutrients contributing to the observed effect [32–34]. Our artificial diet experiments show that the glucosinolate metabolite ITC is responsible for reduced larval development and survival of *T*. *ni*. The detoxification of ITC requires conjugation to glutathione, which is a high energy consumption process and may participate in the trade-offs that we observed (i.e., caterpillar survival, weight reduction, and prolonged development). Furthermore, if the caterpillars feed on a diet that contains higher concentrations of ITC, a higher level of free ITC will not be conjugated, which may lead to the reduced larval performance [26, 27]. Despite this, *T*. *ni* feeds on glucosinolate containing plants, where the caterpillars grow slowly, which may render them more vulnerable to their natural enemies [35, 36]. Interestingly, the impact of ITC was not significant in pupal weight of *T*. *ni*. It is conceivable that exposure to higher levels of ITC triggered a compensatory response in the caterpillars, leading to physiological adjustments that allowed them to achieve comparable pupal weights despite the toxin’s presence. Whether this adjustment comes with a trade-off associated with adult stage will be our next endeavor.

Our data also show that the cellular immunity of *T*. *ni* is negatively affected by the consumption of isothiocyanate (ITC), supporting the ‘immunocompromised host hypothesis’ [8, 14, 37]. Caterpillars that fed on ITC-containing diet had reduced granulocyte and oenocytoid numbers. Interestingly, the number of plasmatocytes increased on diets higher in ITC, which can be correlated with the reduced number of pro-hemocytes. The encapsulation response also requires the function of granulocytes and oenocytoids [38]. Hence, the high plasmatocyte number alone might not provide sufficient protection against parasitoids. This view is supported by the reduced melanization capacity, which is often used as an indicator of immunocompetence in insects [39]. These observations suggest a possible difference between generalist and specialist herbivores. Previous studies on the specialist caterpillars *Plutella xylostella* and *Pieris rapae* have shown that higher glucosinolate concentrations are correlated with enhanced cellular immunity against parasitic wasps at the cost of reduced larval weight and development success [10, 30]. Although, *T*. *ni* caterpillars showed similar trends in weight and development as specialist herbivores like *P*. *xylostella* and *P*. *rapae*, their cellular immunity was negatively affected. This apparent difference could also be due to the presence of other nutrients like carbohydrates, proteins and free amino acids. In *Spodoptera littoralis*, higher protein-to-carbohydrate ratios and the availability of free amino acids were linked to improved antibacterial responses and increased phenoloxidase activity [40–42]. Whether other nutrient factors impact *T*. *ni* immunity and whether a higher protein content and free amino acid diet can rescue the downregulated immune status are topics that require further investigation.

Because the *T*. *ni* cellular immune response was negatively correlated with glucosinolate content, we examined if this was also the case with the humoral immune response. Humoral immunity acts together with cellular immunity to eliminate foreign invaders including parasitoids and pathogenic microbes. Our data indicate that the consumption of a diet containing 2 μmol/g of ITC does elevate the expression level of the AMP-encoding genes *lebocin* and *gallerimycin*, which are produced upon activation of IMD signaling (humoral immune response) [43]. However, the presence of the Gram-negative bacteria *E*. *coli* downregulated the expression level of both AMP genes. Surprisingly, this result is contrary to the known function of *lebocin*, which has been shown to possess antibacterial properties through the agglutination of *E*. *coli* cells in *Manduca sexta* (Lepidoptera: Sphingidae) [44]. Maybe the presence of ITC alone can affect the humoral immune capacity in *T*. *ni* by increasing the transcriptional levels of certain AMP genes such as *lebocin*, but the combined presence of plant and *E*. *coli* toxins may perturbs the activation of the Toll and Immune Deficiency (IMD) pathways that regulate AMP production and activity. Other reasons for this apparent difference could be due to the mode of *E*. *coli* introduction. Previous studies have conducted feeding experiments where the caterpillars were exposed to artificial diet supplemented with bacteria [45, 46], while in our study the bacterial cells were directly injected into the hemocoel. Our approach bypasses the gut immune defense and the potential involvement of gut microbiota, which can provide a priming effect against certain bacteria [4]. On the other hand, although *cecropin* in *T*. *ni* was downregulated by ITC, infection with *E*. *coli* elevates its expression level in comparison to the respective control. This indicates that the detoxification of ITC does suppress *cecropin* expression but fails to perturb the activation pathway in response to infection with non-pathogenic bacteria.

Overall, our data suggest that the presence of plant toxin negatively affects two important life-history traits: caterpillar development and immunity. Whether the observed elevated numbers of plasmatocytes and expression levels of *cecropin* and *gallerimycin* constitute an efficient *T*. *ni* host immune response against parasitoids or microbial pathogens deserves further attention. Nonetheless, the current study is original in terms of demonstrating the impact of glucosinolates on caterpillar immunity. To our knowledge, this is the first report to reveal how the glucosinolate metabolites negatively affect the development as well as modulate cellular and humoral immunity of a generalist caterpillar. This opens a new avenue of investigation to further explore similar effects conferred by toxic plants on the efficiency of various physiological processes of major agricultural insect pests.
